# Development of a Weighted Barite-Free Formate Drilling Mud for Well Construction under Complicated Conditions

**DOI:** 10.3390/polym13244457

**Published:** 2021-12-19

**Authors:** Valentin Morenov, Ekaterina Leusheva, Tianle Liu

**Affiliations:** 1 Department of Oil and Gas, Saint Petersburg Mining University, 199106 Saint Petersburg, Russia; Leusheva_EL@pers.spmi.ru; 2 Faculty of Engineering, China University of Geosciences, Wuhan 430074, China; liutianle2008@163.com

**Keywords:** formate drilling fluid, weighted mud, polymer reagent, inhibition, encapsulation

## Abstract

Construction of oil and gas wells at offshore fields often involves high formation pressure and the presence of swellable clay rocks in the section. In addition, productivity preservation is also an important aspect. For this purpose, it is necessary to reduce the solids content of the drilling mud. The purpose of this work is to develop, improve, and study compositions of weighted drilling muds with low content of solids, on the basis of organic salts of alkali metals and polymers for the construction of wells prone to rock swelling and/or cavings, as well as drilling fluids for drilling-in the formation. In order to achieve the set goal the following is required: Analysis of existing drilling muds of higher density for drilling wells in unstable rock intervals and for drilling in the productive formation; analysis of experience in using drilling systems on the formic acid salts base and substantiation of requirements for flushing fluids during well construction; development and investigation of drilling mud compositions on the formate base; and the evaluation of inhibiting effect of systems containing organic salts, polymer reagents, and calcium carbonate on clay samples. The developed drilling mud is characterized by a high inhibiting ability that allows minimized mud-weighting by the natural solid phase. This reduces the volume of prepared mud and facilitates the regulation of its properties by reducing the dispersion of drilled cuttings; it eliminates problems related to hydration and the swelling of active clay rocks; and stabilizes unstable argillites prone to caving. The low solids content, low filtration rates, and inhibitory nature of the mud allows high stability of the rheological properties of the mud, and preserves oil and gas reservoir productivity under conditions of elevated formation pressure.

## 1. Introduction

### 1.1. Relevance of the Work

For sustainable development of the mineral resources in the Arctic offshore, it is necessary to develop and implement efficient technologies that are suitable for Arctic conditions and safe from an environmental point of view. The construction of oil and gas wells offshore comes with certain problems that hinder efficiency and increase the cost of drilling operations. One of the main challenges before the drilling process is selecting the optimal drilling fluid, which depends on geological conditions, formation pressure, and absorption pressures. It is also necessary to note the system’s ability to preserve the reservoir properties of the productive formation, ensure wellbore stability and integrity; include high zenith angles during the entire drilling interval until its casing; and higher drilling speeds through various sedimentary rocks, such as shale, clay shale, and limestone, etc.

The drilling process is often complicated by the integrity of the borehole walls being compromised by unstable clay deposits (clays, shales, and mudstones). This can result in cavings, rockfalls, borehole constriction, and cavern formation, with cavings becoming more likely with the increase in depth and inclination angle. The increased danger of cavings is caused by the collapse of weakly cemented siltstones and mudstones, which are in contact with solution filtrate; and by plastic flow of montmorillonite clays during osmotic swelling [1]. In order to prevent and eliminate these complications, it is recommended to weight the drilling mud or use systems with low water loss. However, worldwide experience in offshore drilling has shown that such actions do not fully exclude borehole stability disruption [2].

The drilling of easily swollen clays disperses them and produces an increased amount of colloidal particles, resulting in complications, such as pinches, landings, sticking, packing, and reduced efficiency of the flushing system. Consequently, when selecting drilling fluids for drilling in caving zones, the density and water loss of the flushing fluid are not the determining factors. Under these conditions, the choice should be made in favor of the most inert drilling system in contact with unstable formations [3,4].

In order to improve the quality of drilling-in the formation, the solution must be designed to reduce the natural permeability of the productive interval slightly or not at all, in order to provide excellent borehole cleaning and to facilitate further development of the well [5,6]. In addition to being safe and economical to use, drilling-in solutions must be compatible with natural fluids to avoid salt deposition or emulsion formation [1,7]. A suitable non-polluting fluid should form a filter cake on the formation surface, but should not penetrate too deeply into its pore section. The mud filtrate should also prevent swelling of active clay particles within the pore base [8].

When constructing wells in the Arctic offshore, it is advisable to opt for environmentally safe drilling fluids. One of the criteria for the safety of reagents for offshore drilling is the HCMS (Harmonised Mandatory Control Scheme), developed within the framework of the international Oslo–Paris agreement in 2002 [9,10,11]. This assessment is carried out to determine the possible environmental impact of a chemical release at sea in the event of an accident or spill [12]. The research is based on controlling ocean algae growth inhibition and biodegradability in seawater. Only those reagents that pass safety testing are eligible for use in drilling fluids used in offshore drilling and especially under Arctic conditions [13,14].

The drilling of oil and gas wells under offshore conditions has a special focus on the disposal of drilling sludge, which has a negative impact on the environment. Environmental pollution is much greater with the use of hydrocarbon drilling systems than with water-based muds [9,15,16]. Drilling sludge is a mixture of drilling mud and drill cuttings, which, on contact, adsorb on the surface various components of the drilling mud and, as such, remain on the drilling site for a long time, in particular, in sludge pits [17,18].

All of the above-mentioned complications of offshore drilling increase the cost of drilling operations in the Arctic shelf environment. Therefore, search, development, and improvement of environmentally safe drilling muds on the water basis, containing different additives that could give properties similar to those of solutions on the hydrocarbon base, without negative influence on the environment, is a very urgent task.

Existing drilling fluid systems are divided into water-based fluids (WBF), hydrocarbon-based fluids (HBF) and synthetic-based fluids (SBF), and gaseous systems. Factors such as cost, technical characteristics, and environmental impact have a major influence on the choice of solution [19].

The most environmentally friendly are the aqueous solutions that use environmentally friendly materials: Biodegradable polysaccharides, their esters, and clay powders. Particular attention must be paid to the clay inhibitors, surfactants, and lubricating additives required for accident-free operation when using such solutions [9].

A drilling-fluid system is a component of the well construction process that remains in contact with the wellbore throughout the drilling operation. The design of the drilling-fluid system involves the development of its formulation and is carried out so as to perform effectively under the expected conditions in the wellbore [20]. The main functions of drilling fluids and their corresponding properties are shown in Table 1.

In addition to the functions described above, drilling fluids should be selected in such a way as to improve efficiency and safety during the drilling process. 

The aim of the work is to develop, improve, and study compositions of weighted drilling muds with low solid content based on organic salts of alkali metals and polymers for the construction of wells prone to rock swelling and/or caving, as well as for drilling muds for productive drilling-in the formation.

Tasks of the research:

1. Analysis of existing high-density drilling fluids for drilling at unstable intervals and for productive drilling in formations.
2. Analysis of application experience for formic acid salt drilling systems and justification of drilling fluid requirements in well construction.
3. Development and study of formate-based drilling fluid compositions.
4. Assessment of the inhibition effect of systems containing organic salts, polymeric reagents, and calcium carbonate on clay samples.

### 1.2. Brief Literature Review

#### 1.2.1. Inhibition of Clay Particles

Potassium is the most effective of all existing ions in reducing the hydration (inhibiting the hydration process) of clays. The inhibitory nature of potassium is due to the exchange of potassium ions for sodium and/or calcium ions that occurs between clay layers and the fixation of the potassium ion in the crystal lattice of swelling clay minerals [1,21].

Many swelling clays are potassium-selective and absorb potassium ions in preference to sodium ions. In the case of other clays there is a “mass effect”, which means that the exchange of sodium ions for potassium ions is most active when the ratio of potassium ions to sodium ions in the solution exceeds 3:1. The inhibition of the hydration process in clays, in which the majority of the ions are the result of the exchange of potassium ions, occurs due to the low hydration energy of the potassium ions.

The fixation of the potassium ions takes place in the clay scales with a higher-than-average negative charge. This fixation occurs because the potassium ion with a diameter of 2.66 Å enters tightly into the 2.80 Å lattice parameter of the clay structure. This creates ideal conditions for crystalline consolidation. The low hydration energy of the potassium ion also promotes interlayer dehydration, resulting in a dense, tightly held structure. This structure counteracts hydration and cation exchange. When the ion is fixed, the water content in the interlayer space of the clay scale decreases and it becomes very stable [1,9].

Chloropotassium polymer solution systems have been developed to stabilize water-sensitive clays by inhibition created by potassium ions. Due to the inhibitory nature of this solution system, clay hydration is reduced to a minimum, resulting in less cavernosity, less packing on the bit and stabilizer, less caving of clays, and decreased permeability reduction in the productive zone. The system uses potassium chloride salt as the main source of the potassium ions. The system is most effective in the presence of encapsulating polymers. Either polyanionic cellulose (PAC) or partially hydrolyzed polyacrylamide (PHPA) is used for this purpose [22].

These polymers envelop the drilled particles and drilled-in clays, limiting their interaction with water. Due to the fact that clays have varying degrees of sensitivity to water, different concentrations of KCl are required to inhibit different types of clay [23,24].

In addition to KCl, there are many other non-chlorine-containing sources of potassium, such as potassium formate, potassium carbonate, potassium sulphate, potassium acetate, KOH, and others. All of these potassium-containing materials are used to create inhibiting systems for potassium-based drilling fluids [25].

#### 1.2.2. Application of Formate Drilling Muds

Formate brine (sodium, potassium, and cesium salts of formic acid) drilling fluids can successfully compete with HBF and SBF. Formate systems are solutions with low solids content, more environmentally safe than other widely used brine systems, and compatible with formation fluids, which creates prerequisites for increasing mechanical drilling speeds and reducing near-bottomhole zone contamination [26]. However, the systems based on carboxylic acid salts in combination with polymer additives have good inhibiting ability in relation to clay shales, increased thermal stability of polysaccharide reagents, low corrosive activity, and also reduce friction coefficient of drilling fluids (or enhance its lubricating ability).

Low-solid drilling fluids based on formate brines (sodium, potassium, and cesium salts of formic acid) were originally developed to minimize frictional pressure losses when drilling small-diameter or deep wells [27,28]. A number of field and laboratory tests have shown that formate-based formulations cause less damage to the productive horizon than some other conventional fluid formulations and, therefore, have a beneficial effect on well productivity [29,30].

Since the early 1990′s, formate-drilling fluids have been introduced in Canada, the USA, Argentina, Ecuador, Venezuela, North Sea, Middle East, Indonesia, Malaysia, Kazakhstan, and China. Initially, these fluids were developed for drilling high-temperature wells, as demonstrated by successfully drilled wells in the North Sea at temperatures up to 150 °C, in Saudi Arabia at temperatures up to 180 °C, and in the Jidong field in China at temperatures up to 195 °C [28,31]. Despite the fact that formate-drilling systems are quite widespread outside Russia, only in 2017, “AKROS” LLC and PJSC “Gazpromneft” implemented successful field tests using these flushing fluids at the Yuzhno-Priobskoye and Prirazlomnoye (Pechora Sea shelf) fields [32].

For example, Statoil (now Equinor) has drilled seven formate wells in the Kvitebjørn condensate field in the North Sea. The challenges were high pressure (810 bar), high temperature (155 °C), frequent rock interlayering, and a high well slope. The following formulation was developed for these difficult conditions: Cesium formate, potassium formate, reagents for water loss reduction (modified starch and PAC) and bridging/weighting agent (CaCO_3_). The mud retained the following properties during drilling: Plastic viscosity (PV) of 13 to 20 mPa∙s, DSS (YP) of 1.4 to 3.8 Pa and high temperature/high pressure filtration (HT/HP) of 6 to 16 cm^3^. The system based on the formic acid salts mixture resulted in low ECD values and ROP varied greatly from location to location and with different bit types. One well was completed in a record time of 12.7 days, while the average completion time was 20.9 days [33].

Since 2013, more than 120 wells have been drilled in Western Canada using potassium formate-based drilling mud. The first application was at the Montney field, where drilling was initially done with an invert-emulsion mud. In order to increase the ROP of the horizontal section, a solid-free, high-density brine was proposed. With potassium formate-based drilling fluid, not only an increase in ROP (30–50%), but also an increase in average bit life compared to HBF, was observed. This brine demonstrated excellent performance in the horizontal section due to its near-Newtonian rheology, maintaining a turbulent regime at almost any pumping rate. At the Montney field area, more than 45 sidetracks have since been drilled with potassium formate with reduced drilling times [34].

Byrne M., Patey I., Liz G., Downs J., Turner J. in [27] highlighted the following benefits of using formic acid salt systems in the construction of deep wells and small diameter wells: Maintaining the carrying capacity of solids at high temperatures; prevention of solids settling at high temperatures; minimum circulating pressure loss; low probability of differential sticking (formate-based fluids form a thin impermeable smooth filtration crust); low ECD values in long wells and small diameter wells; compatibility with formation fluids and, as a result, prevention of reservoir damage; compatibility with completion equipment and elastomers; and environmental safety and biodegradability.

Subsequent works have shown that these brines, because of their high density and low corrosivity, are also ideal completion and packer fluids. Formate brines are environmentally friendly and compatible with reservoir fluids, and also provide inhibition of clay shale [31].

Formate systems are highly effective in controlling wellbore instability associated with complex lithologies (shale, gypsum, and salt formations). Laboratory investigations have shown that shale integrity and durability are actually improved when exposed to formate-based fluids. Potassium formate is the strongest inhibitor of the monovalent formic acid salts and promotes shale drilling by reducing swelling pressure, shale water content, and pore pressure.

Formates increase wellbore stability by the following mechanisms: Reduction of pore pressure due to low water activity index and creation of reverse osmosis; reduction of swelling pressure (K+ is more effective than Na+ and divalent cations); and reduced permeation of the filtrate (Darcy′s law)—the filtrate is more viscous than other salts [35].

Research [24] confirms the compatibility of formates with a range of formation materials after a series of laboratory formation damage tests carried out under simulated formation conditions with real rock samples. Unlike polymer and KCl/glycol systems, potassium formate brine has a density of 1.57 g/cm^3^ without the addition of weighting agents, such as barite or ilmenite, while KCl-based brine will not exceed 1.15 g/cm^3^, meaning that weighting material must be added to achieve the desired density. Once such a solution penetrates the productive horizon, solids in large quantities will damage the formation, whereas the potassium formate brine is a minimally damaging system, free of solids.

## 2. Methodology and Equipment

Experimental research into the development of the formulations of drilling fluids based on formic acid salts containing polymeric reagents and marble aggregate was conducted in the Drilling Fluids laboratory at Saint Petersburg Mining University.

The main drilling fluid properties are density (specific gravity), specific viscosity (SV), plastic viscosity (PV), dynamic shear stress (DSS), static shear stress (SSS), filtration index (F), inhibiting capacity of drilling fluid, and value of hydrogen index (pH). 

The inhibiting capacity of drilling fluid was evaluated using the Fann Linear Swell meter 2100 (Houston, TX, USA). A lever scale (mud balance Fann) was used to measure the density of the drilling fluid [36]. The specific viscosity is determined by flowing a certain amount of drilling fluid through a Marsh viscometer. The measuring cup has a volume of 1 L; there is a mark at 0.946 L (1 quart) [37,38]. A Fann model 35A 6-speed rotary viscometer was used to determine the rheological properties of the designed and tested drilling fluids. The compositions of the formate-drilling fluids were tested by rotating the outer cylinder at 600, 300, 200, 100, 6, and 3 rpm.

Plastic viscosity in centipoise (cP) or millipascal on second (mPa∙s) is calculated as the difference between the reading of the Fann viscometer (θ) at 600 and 300 rpm:

PV = θ_600_ − θ_300_ [cP]

where θ_600_ and θ_300_—the rotation angles of the viscosimeter scale at sleeve speeds of 600 and 300 min^−1^, respectively, in degrees.

The dynamic shear stress in lb/100 ft^2^ is calculated from the Fann viscometer data using the formula:

YP = θ_300_ − PV [lb/100 ft^2^] or

YP = (θ_300_ − PV) 4.48 [dPa]

where θ_300_—instrument reading at 300 rpm, PV—plastic viscosity; 4.48—conversion factor for lb/100 ft^2^ into dPa.

According to the API standard, Gel_10sec/10min_ system values are obtained on a rotary viscometer, at a speed of 3 rpm after 10 sec and 10 min of resting liquid [36,37].

Using the API methodology (Fann НРНТ filter press was used in this study), drilling fluid engineers estimate the filtration rate of the mud at ambient temperature and at 100 psi (0.69 MPa). The analysis consists of determining the flow velocity of the fluid through the filter paper. The result is the volume of filtrate produced (mL) in 30 min [37,38].

The pH is determined by the following methods: Either colorimetric, i.e., by comparing the color of the indicator paper moistened with a standard solution, or by using a pH-meter.

**Figure 1:**
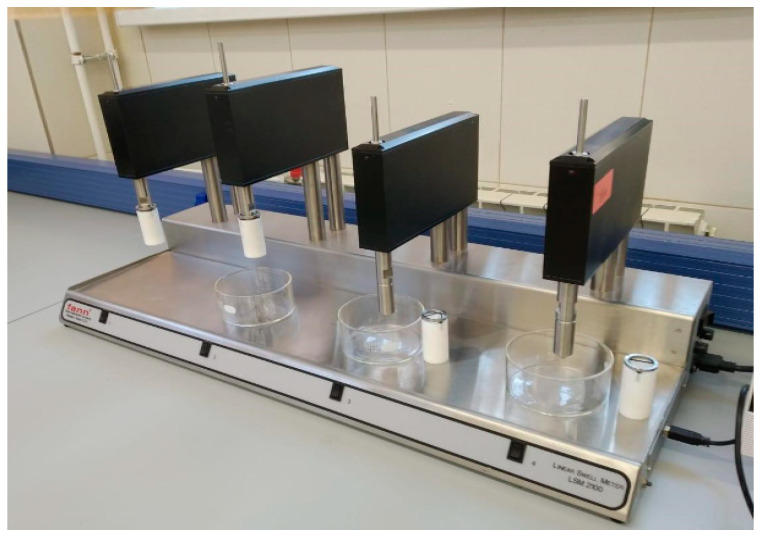
Linear Swell meter Fann 2100.

To predict and solve problems with unstable rocks encountered during well construction, the Fann Linear Swell meter 2100 (LSM) is used to measure the degree of swelling for a clay sample by soaking it in a test fluid. The model 2100 Linear Swell meter includes an automatic measuring system, sealing unit, and software. LSM software records the measurements and displays the results as a real-time graph, showing the percentage of swelling as a function of time. An image of the instrument with which the inhibition capacity of the solution can be assessed is shown in Figure 1.

The Fann twin-chamber manual hydraulic compactor is used to compact clay material samples. “Tablets” are formed over several hours under a constant pressure of 8 MPa produced by a hand pump. The clay is placed in a cylindrical mold (rod chamber), which is connected to the compaction cell (hydraulic cylinder), where compaction takes place.

The assessment of inhibition capacity is based on determining the percentage increase in height of the sample over a certain period of time, the increase in which is caused by swelling pressure. The material used to make the “tablets” of the clay sample is “PMBM” bentonite, which is unprocessed with polymers. A total of 20 g of clay are required to create one tablet, which is compressed on a Fann compactor for 2 h at a constant pressure of 8 MPa. Accordingly, the smaller the increment in height, the better the inhibiting capacity of the liquid [39].

## 3. Investigations and Results

The first stage in the development of formate-based high-density drilling compositions was to work out the preparation of the base mud. It was decided to use the formulation developed by the oilfield service company “Akros” as the basis.

The base mud is based on HCOONa (sodium formate) and HCOOK (potassium formate), which is also an inhibitor (reagents supplied by Saint Petersburg Mining University, Saint Petersburg, Russia). A biopolymer, xanthan gum, is added to the solution as a structure builder and starch and VPRG (polyacrylonitrile) are used to reduce the filtration index. K_2_CO_3_ (potassium carbonate) is added to maintain the correct pH level. The bridging agent is CaCO_3_ MEX-CARB (calcium carbonate) and the defoamer is octanol.

**Figure 2:**
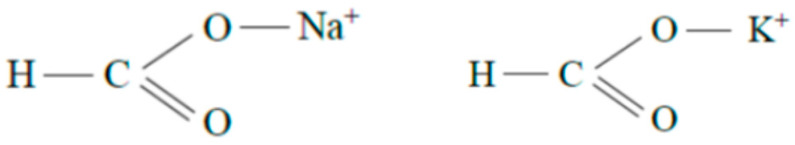
Sodium formate and potassium formate.

Formates of alkali metal salts are soluble in water and form brines of rather high density. Two formic acid salts, sodium formate (HCOONa) and potassium formate (HCOOK), are used in this work; the molecular structures of these compounds are shown in Figure 2.

Formate brine systems provide a wide range of densities. Sodium formate is the least soluble in water and can produce a brine density of 1.33 g/cm^3^. Potassium formate is more soluble, with a maximum brine density of about 1.57 g/cm^3^. In order to prepare a brine of potassium formate or sodium formate, the required amount of salt must be dissolved in a certain amount of water, depending on the density of the solution required.

**Figure 3:**
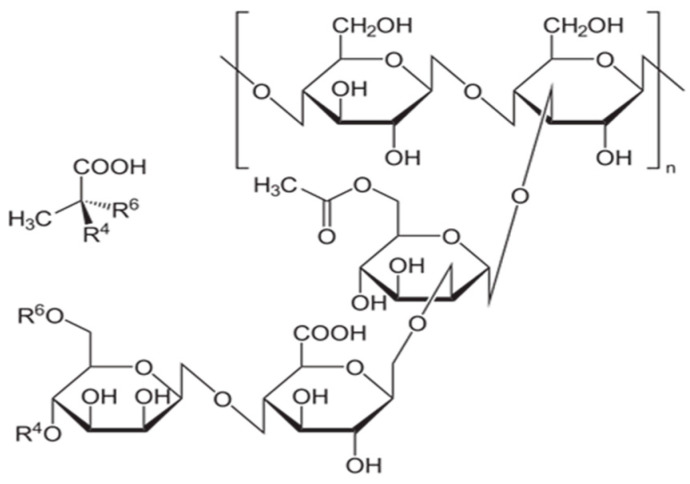
Molecular structure of xanthan gum.

The biopolymer “Bioxan” by Polycell (xanthan gum), a natural highly branched polysaccharide with an extremely high molecular mass, which gives the system pseudoplastic properties, is used as a structure builder in the studied solutions. The molecular structure is shown in Figure 3.

Xanthan helps to increase viscosity in drilling fluids due to its long branched structure and relatively weak hydrogen bonds of side groups [40]. Functional groups are represented by hydroxyl (-OH), carboxyl (-COH), carbonyl (C=O) and other groups, which give this polymer its thickening properties [6,41].

The synthetic polymers, polyacrylonitrile (HIPAN or VPRG), are added to the muds to reduce the filtration index of the drilling systems studied, and partially hydrolyzed polyacrylamide (PHPA) is used for the further research and development of new compositions.

By adsorbing on the solids particles, creating insulating layers, these reagents prevent peptization, flocculate the solids particles, and generally create an inhibition effect [22]. Synthetic polymers also influence the rheological characteristics of the drilling fluid and reduce hydraulic resistance during circulation [42].

Polyacrylonatrile reduces the solids effect (increase in mud volume, density, and viscosity due to the transfer of drilled clay rock into the mud) during drilling and enhances the lubrication and anti-sticking properties of the drilling fluid. When interacting with clay particles through chelate bonds, strong polymer-clay structures and polymer membranes are formed, contributing to reduced rock swelling.

**Figure 4:**
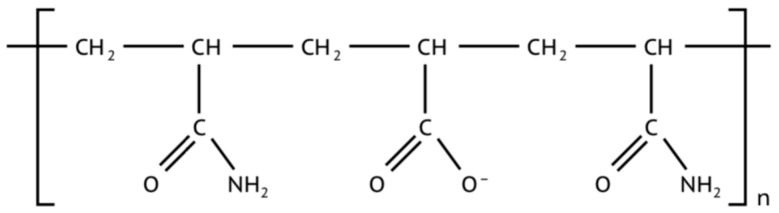
Molecular structure of partially hydrolyzed polyacrylamide.

PHPA is a copolymer containing two or more different types of monomers (acrylate and acrylamide). The two monomers bond together to form a linear carbon chain. Molecular structure of PHPA is shown in Figure 4. However, acrylamide is a water insoluble compound, so copolymerization with sodium acrylate is necessary to achieve solubility. Carboxylic group in the polyacrylate facilitates the conversion of PHPA into an anionic polymer. Strong carbon–carbon bonding makes PHPA thermally stable and resistant to bacterial decomposition.

Inhibition occurs through the following mechanisms:

(1) Encapsulation: РHPA encapsulates the clay scales and the wellbore due to the fact that the polyacrylate has too high an affinity to the positive clay edges. Because РHPA has a long polymer chain and a high molecular mass, it bonds to multiple sites along the borehole, i.e., the anionic -COO groups are attracted to the positively charged particle edges, helping to form a protective coating along the borehole that prevents the clay from coming into contact with water. The encapsulation process also prevents water from entering the interlayer structure of the clay.
(2) Increasing the viscosity of the filtrate (thickening of the aqueous phase): This slows down penetration of the liquid into the interlayer structure of the clay.
(3) Adsorption (the taking-up of free water by the polymer): This reduces the amount of water “available” for hydration of the clay [9].

In this case, if the polymer consisted only of anionic carboxylic functional groups, which have too high affinity to the positive charges of the clay particles, the forces of attraction on contact would separate the clay particles from each other, which would lead to dispersion of the clay. Amide group (-NH_2_) allows the polyacrylate groups to distribute some distance from the cations on the clay particles. The same effect can be observed for the sludge. The polymer helps to isolate the sludge particles, which allows for more efficient cleaning of the solution on the surface [40].

Efficiency of a particular polymer depends on the ionic strength of the solution. Each of the polymers has its own effect on changing the technological properties of the polymers. Therefore, finding the right concentration of a particular polymer or a combination of polymers is a challenging and relevant task in order to increase the efficiency of the solution.

The rheological and filtration parameters of the prepared base mud were determined in the laboratory using the equipment presented earlier. Results obtained are presented in Table 2.

In the second stage, the basic formulation was improved in order to optimize rheological and filtration characteristics, increase the inhibiting capacity of the drilling fluid, and improve the quality of wellbore cleaning by using hydrolyzed polyacrylamide (PHPA) of different molecular mass (from 12 M to 27 M) as a substitute for VPRG. The concentration of PHPA in each of the solutions is 0.5 g/L. When the concentration of PHPA was increased up to 2 g/L, the Weissenberg effect was observed (the mud was coiled up on the stirring element), so it was decided to reduce the amount of PHPA. The main parameters of the developed drilling fluids are presented in Table 3.

### Evaluation of the Inhibiting Capacity of Developed Formate-Based Drilling Fluids

Numerous investigations prove that the problem of improving wellbore stability in unstable clay deposits can be solved by incorporating electrolytes and polymers into the solution used.

Inhibiting the capacity of a flushing fluid refers to its ability to prevent or slow down deformation processes in the near-wellbore space. Since swelling and dispersing are continuous processes, the use of encapsulating polymeric reagents is justified because a polymer of this type will slow down both processes [22,25,43].

To assess the inhibiting capacity, a basic drilling fluid treated with VPRG and four fluids with the addition of PHPA are tested. 

PHPA performs the following functions in the drilling fluid: It reduces the filtration of drilling mud; acts as an encapsulant, i.e., penetrates cuttings and clay particles by means of ion attraction and hydrogen bonding; acts as a clay inhibitor, i.e., prevents or slows down hydration, or disperses of mud; and prevents caving and erosion of clays.

Formulations of the highly mineralized formate systems investigated contain two reagents with inhibitory properties: Potassium formate and PHPA. Potassium formate is the strongest inhibitor of the monovalent formic acid salts. Due to the high concentration of K+ ions, which reduces the intrusion zone of the filtrate into the formation, it is effective in suppressing clay hydration. Potassium formate contributes to the stability of the borehole by the following mechanisms: Reduction of pore pressure P_pore_ due to low water activity index and creation of reverse osmosis; reduction of swelling pressure P_swell_ (K+ is more effective than Na+ and divalent cations); and reduced permeation of the filtrate (Darcy′s law)—the filtrate has a higher viscosity than other salts.

**Figure 5:**
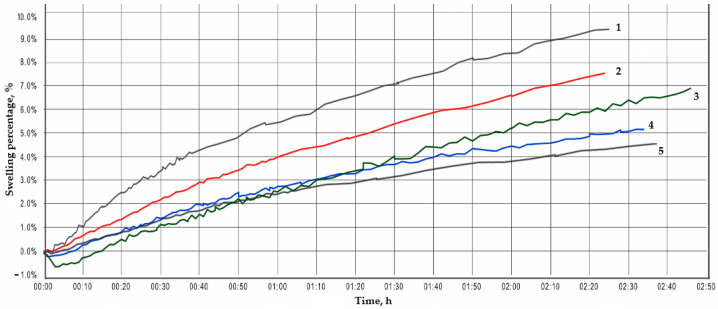
Swelling curves of clay samples in the investigated solutions. 1—“VPRG”; 2—“РНРА 12”; 3—“РНРА 15”; 4—“РНРА 20”; 5—“РНРА 27”.

In order to predict and solve problems with unstable rocks encountered during well construction, the Fann Linear Swell Meter (LSM 2100) is used to measure the degree of swelling of a clay sample by soaking it in the test fluid. Figure 5 shows the time dependence (h) of the swelling percentage of the clay sample in different solutions obtained with the LSM 2100.

Analysis of inhibition ability was conducted for 2 h and 30 min in order to establish the dynamics of swelling; with further increase of time, the dependence of the percentage increase in height on time will have a linear character. In order to exclude the probability of random error, the experiment was conducted simultaneously on three cells for each investigated drilling mud. For clarity, the obtained dependencies are summarized in the general graph.

## 4. Discussion

A basic composition of a highly mineralized clayless mud, with a density of 1.45 g/cm^3^ without barite and with a minimum of calcium carbonate, has been obtained. The high density of the mud is achieved by formic acid salts. To obtain a thin, smooth filter cake with minimum permeability, chalk of different fractional composition is added to the solution.

Developed formulation, based on the results obtained, has several benefits. Replacing VPRG with РНРА decreases the filtration rate of the solution, with the increasing molecular mass of РНРА the filtration rate decreases, which helps to reduce the probability of differential sticking. In addition, this solution promotes the formation of a thin, impermeable filter cake that will prevent filtrate from penetrating the formation. Increasing the molecular mass of the РНРА results in an increased DSS, but has no effect on plastic viscosity.

When measuring the degree of swelling of the clay sample, its maximum expansion in 2 h and 20 min in the solution treated with VPRG is 9.2%, while in the solution containing РНРА with a molecular mass of 12 M is 7.3%. Height of the sample in the “РНРА 15” solution increases by 6% and in the “РНРА 20” height of the sample increases by 5%.

Based on performed investigations, the best inhibiting ability is in the formate-based mud, which contains 0.1% РНРА with a molecular mass of 27 M, because it shows a minimal change in the height of the clay sample (4.2%), compared with all the studied solutions.

## 5. Conclusions

Thus, drilling fluid formulations treated with РНРА-polymer used as a clay hydration inhibitor provide a system close to the inhibiting capacity of HBF. At the same time, acrylic polymer with the highest molecular mass among those selected for investigations (27 M) must be used to provide the best inhibition. Experimental results show that the swelling inhibition mechanism increases with increasing РНРА molecular mass. Phenomenon of synergistic effect by treating the studied solutions with potassium formate and РНРА of different molecular mass are experimentally (using linear clay swelling tester) confirmed. Correspondingly, НСООК and РНРА mutually complement and enhance the inhibiting effect of the washing fluid, which results in a decrease in time for the liquidation of complications and increase of mechanical ROP. Phenomenon of synergistic effect is concluded based on the fact that the swelling of the “pill” in the medium of the solution combining potassium formate and РНРА is 2.2 times lower than in the medium of the fluid with potassium formate and VPRG.

It should be noted that the developed drilling mud is characterized by high inhibiting capacity, which allows a minimizing solids effect at the expense of natural (drilled out) solid phase. This reduces volumes of prepared mud and facilitates the regulation of its properties, reducing dispersion of drilled out mud; eliminates problems related to hydration and the swelling of active, clay rocks; and stabilizes unstable and caving-prone argillites. Low solids content, low filtration rates, and the inhibitory nature of the mud allows high stability of the rheological properties of the mud, and preserves oil and gas reservoir productivity under conditions of elevated formation pressure.

## Figures and Tables

**Table 1:** Functions and corresponding properties of drilling fluids.

Function of Drilling Fluid	Corresponding Property of Drilling Fluid
Removing the cuttings from under the bit, transporting it to the wellhead	Dynamic shear stress, apparent viscosity, flow velocity, static shear stress
Preventing fluid influx into the wellbore	Density
Maintaining the wellbore in a stable condition	Density, interaction with clays (inhibition)
Cooling and lubrication of drill string and bit	Density, flow velocity
Energy transfer from pumps to downhole motors, turbo-drills and displacement motors	Flow velocity, density, viscosity

**Table 2:** Parameters of the formate base mud.

Parameter	Base Mud
Density, g/cm^3^	1.45
Specific viscosity, s/quarter	42
600 rpm	49
300 rpm	30
200 rpm	24
100 rpm	15
6 rpm	5
3 rpm	3
Plastic viscosity, mPa∙s	19
DSS, Pa	5.3
SSS (10 s/10 min), Pa	2.4/3.8
Filtration, mL/30 min	3.8
рН	10

**Table 3:** Parameters of the formate-based mud formulations developed.

Parameter	Solution “РНРА 12”	Solution “РНРА 15”	Solution “РНРА 20”	Solution “РНРА 27”
Density, g/cm^3^	1.45	1.45	1.45	1.45
Specific viscosity, s/quarter	40	40	41	42
600 rpm	43	46	49	52
300 rpm	27	30	33	36
200 rpm	21	24	26	2
100 rpm	14	17	18	17
6 rpm	5	5	5	5
3 rpm	3	3	3	3
Plastic viscosity, mPa∙s	16	16	16	16
DSS, Pa	5.3	6.7	8.1	9.6
SSS (10 s/10 min), Pa	2.4/3.8	2.4/3.8	2.4/3.8	2.4/3.8
Filtration, mL/30 min	3	2.7	2.6	2.2
рН	10	10	10	10

## Data Availability

The data presented in this study are available on request from the corresponding author. The data are not publicly available due to its storage in private networks.

## Abbreviations

The following abbreviations are used in this manuscript:

WBF	Water-based fluid
HBF	Hydrocarbon-based fluid
SBF	Synthetic-based fluid
ECD	Equivalent circulation density
ROP	Rate of penetration
PHPA	Partially hydrolyzed polyacrylamide
PV	Plastic viscosity
SV	Specific viscosity
DSS	Dynamic shear stress
SSS	Static shear stress
